# Development of a Novel KCNN4-Related ceRNA Network and Prognostic Model for Renal Clear Cell Carcinoma

**DOI:** 10.1155/2023/2533992

**Published:** 2023-01-24

**Authors:** Hengtao Bu, Qiang Song, Jiexiu Zhang, Yuang Wei, Bianjiang Liu

**Affiliations:** Department of Urology, The First Affiliated Hospital of Nanjing Medical University, Nanjing 210029, China

## Abstract

**Background:**

Clear cell renal cell carcinoma (ccRCC) accounts for more than 80% of renal cell carcinomas. Yet, it has not been fully understood about the derivation and progression of the tumor, as well as the long-term benefits from multimodality therapy. Therefore, reliable and applicable molecular markers are urgently needed for the prediction of diagnosis and prognosis of ccRCC patients.

**Methods:**

Genetic and clinical information of 533 ccRCC patients from The Cancer Genome Atlas database was collected for comprehensive bioinformatic analyses. UALCAN was used to detect gene expression in paired tumor samples. Two data sets from Gene Expression Omnibus database were analyzed to identify differentially expressed genes (DEGs), and Gene Set Enrichment Analysis was applied for the functional enrichment of DEGs. Tumor Immune Single Cell Hub and Tumor IMmune Estimation Resource databases were separately used for analyses of single-immune cell and immune cell infiltration. Encyclopedia of RNA Interactomes database was explored to predict targeted microRNAs (miRNAs) and corresponding long non-coding RNAs (lncRNAs). Cox regression analysis was performed for the construction of risk signature and prognosis model. Finally, quantitative real-time polymerase chain reaction and western blot were conducted for KCNN4 expression detection in cell lines and clinical samples. Small interfering RNA was employed to knock down KCNN4, and corresponding functional experiments were conducted on ccRCC cells as well.

**Results:**

KCNN4 showed elevated expression in tumors and prominent clinical correlation in ccRCC. In total, 41 KCNN4-related genes were enriched, and Gene Ontology and Kyoto Encyclopedia of Genes and Genomes analyses showed they were intimately related to immune-related signaling pathways. Spearman's analysis revealed the significantly positive correlation of KCNN4 with immune cell infiltration. By integrating hub miRNA-let-7e-5p and four critical lncRNA, a competitive endogenous RNA network-based risk signature was constructed. The prognosis model derived from it showed considerable predictive value for survival of ccRCC patients. Finally, in vitro experiments confirmed the remarkable tumor-promoting role of KCNN4 in ccRCC cells.

**Conclusion:**

KCNN4 significantly affected the immune status of tumor microenvironment and immunotherapy elements, through which it promoted tumor progression in ccRCC, and it could be a potential biomarker for prognosis and immunotherapy effects of ccRCC patients.

## 1. Introduction

Renal cell carcinoma (RCC), originating from the renal tubular epithelium, accounts for about 2% of adult malignancies worldwide [1]. According to the global cancer statistics, there were approximately 403,262 new RCC cases and 175,098 RCC-related deaths in 2018 [2], of which 80–90% are clear cell renal cell carcinomas (ccRCC) [3]. In recent years, the incidence of ccRCC has gradually increased, with the age at diagnosis continuing to fall [4]. Due to the hidden symptoms and lack of applicable markers for diagnosis, approximately 30% patients have distant metastasis at the time of diagnosis [5, 6]. ccRCC patients in early stage normally benefit from timely surgical treatment, but for advanced tumors, the 5-year survival rate is only 23% [7]. Although multiple therapies (e.g., small molecular targeted drugs and immunotherapy) are available for ccRCC patients, improvements in disease-free survival (DFS) and overall survival (OS) are far from satisfying [8]. Considering the limited tools of diagnosis and methods of treatment, novel reliable biomarkers are urgently needed for early diagnosis and long-term prognosis prediction.

KCNN4 (also known as IKCa, SK4, or KCa3.1) encodes the protein which belongs to the calcium-activated potassium channel subfamily. Gardos first reported it and described its function in 1958 [9]. Activated by membrane hyperpolarization, KCNN4 is widely expressed in excitable and non-excitable cells and plays a major role in many cell functions. Forty years later, it was successfully cloned, and the result showed it is a tetrameric membrane protein, each subunit consists of six transmembrane segments [10]. In previous study, KCNN4 is strongly associated with unfavorable clinicopathological features and poorer survival. It was induced by the transcription factor AP-1 and mediates the calcium/MET/AKT axis to promote the proliferation and migration of pancreatic ductal adenocarcinoma [11]. It induces multiple chemoresistance in breast cancer by regulating BCL2A1 [12]. It is even involved in the infiltrative behavior of glioblastoma in vivo [13]. Besides, aberrant expression of KCNN4 has been closely associated with papillary thyroid cancer, hepatocellular carcinoma, and colorectal cancer [14–16].

In the present study, we performed comprehensive bioinformatics analyses to study the clinical correlation and immune association of KCNN4 in ccRCC. We also asked for KCNN4-related competitive endogenous RNA (ce-RNA) network as a risk signature to establish the prognosis model for ccRCC patients. This may provide novel insights into improving the diagnosis and prognosis of patients with renal clear cell carcinoma.

## 2. Materials and Methods

### 2.1. Renal Clear Cell Carcinoma Data Sourcing and Preprocessing

The genetic information of paired or unpaired samples from 533 ccRCC patients, and their clinical characteristics were obtained from The Cancer Genome Atlas (TCGA) database (https://cancergenome.nih.gov/). Data analysis was performed using R (version 3.6.1) and the R Bioconductor software package. Before further analysis, the expression data were normalized to the transcripts per kilobase million values.

### 2.2. UALCAN Database and GDSC Database

UALCAN (http://ualcan.path.uab.edu/) is an interactive web portal for convenient analysis of gene expression data in various tumor samples from TCGA database. Here, UALCAN was used to investigate the expression level of KCNN4 in tumor and normal samples, as well as in different tumor grades, cancer stages, subtypes, and nodal metastasis. Meantime, expression of related microRNAs (miRNAs) and long non-coding RNAs (lncRNAs) in paired ccRCC samples were also detected using UALCAN. Genomics of Drug Sensitivity in Cancer (GDSC) database (https://www.cancerrxgene.org) was used to predict the relationship between KCNN4 and clinical drug sensitivity.

### 2.3. Identification of Differentially Expressed Genes

Genes significantly correlated with KCNN4 expression in ccRCC samples were collected from TCGA database. Only those with a correlation coefficient |cor| > 0.5 and a *p* < 0.05 were selected. The “limma” package was integrated into the R software to identify the differentially expressed genes (DEGs) between the high-KCNN4 group and the low-KCNN4 group, which was divided according to median expression of KCNN4. Genes with adjusted *p* < 0.05 and |log2(FC)| > 1.0 were considered as DEGs. Ultimately, the heatmaps were used to show the top 26 differentially expressed KCNN4-related genes in two data sets.

### 2.4. Functional Enrichment Analyses on KCNN4-Related Genes

The Gene Set Enrichment Analysis (GSEA) was conducted on DEGs using clusterProfiler package in the R software. The standard gene sets of gmt file C2 (Kyoto Encyclopedia of Genes and Genomes, KEGG) and C5 (Gene Ontology, GO) were downloaded from the GSEA website (http://www.gsea-msigdb.org/gsea/msigdb) for reference. Then, the analyses on KEGG pathways and GO were conducted on KCNN4-related genes. The latter included three functional categories: biological process (BP), cellular component (CC), and molecular function (MF). After the analysis, only terms with *p* < 0.05 were considered statistically significant.

### 2.5. Tumor Immune Single-Cell Hub Database

Tumor Immune Single-cell Hub (TISCH; http://tisch.comp-genomics.org/) is a scRNA-seq database focusing on tumor microenvironment (TME). TISCH provides detailed cell-type annotation at the single-cell level, enabling the exploration of TME across different cancer types. Here, TISCH was applied to analyze three data sets from Gene Expression Omnibus (GEO) database. KCNN4 distribution in diverse single immune cell and cell proportion were detected accordingly.

### 2.6. Immune Cell Infiltration Analysis

The Tumor IMmune Estimation Resource (TIMER) database (http://timer.cistrome.org/) is a comprehensive resource for systematical analysis of immune infiltrates across diverse cancer types. The estimated immune infiltrating abundances makes it possible to interactively explore the relationship between immune infiltration and various prognostic factors. Here, the TIMER database was used to evaluate the correlation between KCNN4 and the levels of representative immune cell infiltration markers, including B cells, CD4+ T cells, CD8+ T cells, and regulatory T cells.

### 2.7. Prediction of Targeted miRNAs and lncRNAs

The Encyclopedia of RNA Interactomes (ENCORI) database (also known as starBase previously, https://starbase.sysu.edu.cn/) mainly focuses on miRNA-target interactions, which identifies more than 2.9 million miRNA–messenger RNA (mRNA) and 4.1 million miRNA–non-coding RNA (ncRNA) interactions from multi-dimensional sequencing data. In this study, ENCORI was used to predict potential miRNAs that targeted KCNN4 and corresponding competitive lncRNAs to KCNN4. In total, 41 miRNA and 12 lncRNA were picked out with the adjusted *p* < 0.05.

### 2.8. Construction of the Gene Signature and DCA

The univariate Cox regression analysis was conducted on all lncRNAs, and their hazard ratio (HR) number was calculated accordingly. Then, they were subjected to the Least Absolute Shrinkage and Selection Operator (LASSO) Cox regression analysis to calculate the optimal weighting coefficient and build the risk model with the “glmnet” package (https://glmnet.stanford.edu/). At last, four of the lncRNA, along with miRNA and KCNN4, were included into the risk model. By dividing ccRCC patients from the training cohort into high- and low-risk groups upon the optimal cutoff of the risk score, we analyzed the survival rate for each patient using the method of Kaplan–Meier (KM) survival. The risk score for the signature was evaluated by using the following formula:

(1) $$\begin{array}{c} \text{Risk score}=\overset{n}{\underset{i=1}{\sum }}{\text{Coef}}_{i}\times x_{i,} \end{array}$$

where Coef_*i*_ and *x*_*i*_ are, respectively, the coefficient and the *z*-score transformed relative expression value of each selected gene. Decision curve analysis (DCA) can assess the utility of models for decision making, we built DCA using the R package: “ggDCA”.

### 2.9. Quantitative Real-Time PCR and Western Blot

As previously described [17], total RNA was isolated from Caki-1 cells by TRIzol Reagent (Thermo Fisher Scientific, Waltham, MA, USA) and reversely transcribed into complementary DNAs using the Reverse Transcription Kit (Thermo Fisher Scientific) following the manufacturer's protocol. Then, the quantitative real-time polymerase chain reaction (qRT-PCR) was conducted on a LightCycler 480 II (Roche Diagnostics, Basel, Switzerland) instrument using the SYBR-Green Master Kit (Vazyme, Nanjing, China). The primers used to amplify KCNN4 were purchased from MasterBio (Beijing, China), designed as 5′- GCAGAGGAGTAAGAAGGTGGAA-3′ (forward) and 5′-TGGCAGGAACTGGCATTG-3′ (reverse). The primers for *β*-actin were 5′-ACTGGAACGGTGAAGGTGAC-3′ (forward) and 5′-AGAGAAGTGGGGTGGCTTTT-3′ (reverse). Each qRT-PCR was performed in triplicate, and *β*-actin was utilized as a control to normalize KCNN4 expression. The primers used to amplify miRNA hsa-let-7e-5p was designed by the Vazyme miRNA primer design software (version 1.01) and synthesized by Tsingke Biotechnology (Beijing, China). The forward primer sequence is 5′-CGCGTGAGGTAGGAGGTTGT-3′; the reverse primer sequence is 5′-AGTGCAGGGTCCGAGGTATT-3′. The stem-loop sequence is 5′-GTCGTATCCAGTGCAGGGTCCGAGGTATTCGCACTGGATACGACAACTAT-3′. The primer sequence of U6 (internal reference) is as follows: forward primer sequence: 5′-CTCGCTTCGGCAGCACA-3′; reverse primer sequence: 5′-AACGCTTCACGAATTTGCGT-3′.

Similarly, total proteins were extracted from Caki-1 cells using radioimmunoprecipitation assay buffer. Proteins were separated by 10% sodium dodecyl sulfate–polyacrylamide gel electrophoresis and transferred to a polyvinylidene difluoride membrane. After blocked within skim milk for 2 hours, the membranes were incubated overnight with the primary antibody (Cell Signaling Technology, Danvers, MA, United States) specifically against KCNN4 at 4°C. Then, the secondary antibody anti-mouse IgG (Cell Signaling Technology) was used to incubate the membranes for 2 hours. Glyceraldehyde 3-phosphate dehydrogenase expression was used as a loading control. Finally, the protein bands were visualized with an enhanced chemiluminescence detection system (Thermo Fisher Scientific, Rochester, NY, USA).

### 2.10. KCNN4 Silencing Using Small Interference RNA

RNA interference of KCNN4 was accomplished using small interfering RNAs (siRNA; HanBio, Shanghai, China). The sequences of siRNAs used were as follows: siRNA1: 5′-GCACCUUUCAGACACACUUTT-3′ (forward); 5′-AAGUGUGUCUGAAAGGUGCTT-3′ (reverse). siRNA2:5′-GGGAACAAGUGAACUCCAUTT-3′ (forward); 5′-AUGGAGUUCACUUGUUCCCTT-3′ (reverse). Caki-1 cells were seeded onto 6-well plate, and those at logarithmic growth phase were transfected with siRNA using Lipofectamine 3000 (Invitrogen, Thermo Fisher Scientific). Then, qRT-PCR and western blot were used to evaluate the efficiency of silencing.

### 2.11. In Vitro Proliferation, Migration, and Invasion Assays

Cell Counting Kit-8 (CCK8) assay was used to measure the proliferative ability of Caki-1 cells. First, the cells were resuspended, diluted, and seeded onto 96-well plates (1500/well), cultured in 5% CO_2_ and 37°C overnight. At the time of 24, 48, 72, and 96 hours, 10 *μ*l of CCK8 reagent (Solarbio, Beijing, China) was added to each well and incubated for 1 hour. After that, the absorbances at 450 nm for knockdown and control groups were measured accordingly. The colony formation experiment was carried out in a 6-well plate. The KCNN4-knockdown Caki-1 cells were planted in a 6-well plate with 2000 cells per well. After 9 days of culture, they were fixed and stained and photographed for counting.

For the measurement of migration and invasion capability, transwell assay and wound healing assay were performed, respectively. A total of 1 × 10^4^ cells were seeded into the upper chamber (Corning Incorporated, New York, USA) with 200 *μ*l serum-free 1640 medium. The lower chambers were filled with 1640 containing 10% fetal bovine serum. After incubated at 37°C for 24 hours, cells on the lower surface of the membrane were fixed in 0.4% paraformaldehyde and stained with 0.1% crystal violet dye for 20 minutes at room temperature. Finally, after washing with Phosphate buffer solution (PBS), cells were imaged in five randomly selected fields under a light microscope (Olympus Corporation, Tokyo, Japan) at 100× magnification.

### 2.12. Statistical Analysis

All analyses were performed using the R software (version 3.6.1), the GraphPad Prism 9.0, and the ImageJ software. The Wilcoxon test was used to compare the expression of genes in different clinical and pathological characteristics of ccRCC samples in the TCGA and GEO database. The Spearman's correlation analysis was used to identify correlations between various immune cell infiltration markers and KCNN4. Univariate and multivariate Cox regression analyses were sequentially used in the construction of risk signature. KM method was used to assess the distinction on OS or DFS according to specific grouping. All statistical tests were two-sided, and a *p* < 0.05 was considered statistically significant.

## 3. Results

### 3.1. Elevated Expression and Remarkable Clinical Correlation of KCNN4 in ccRCC

**Figure 1:**
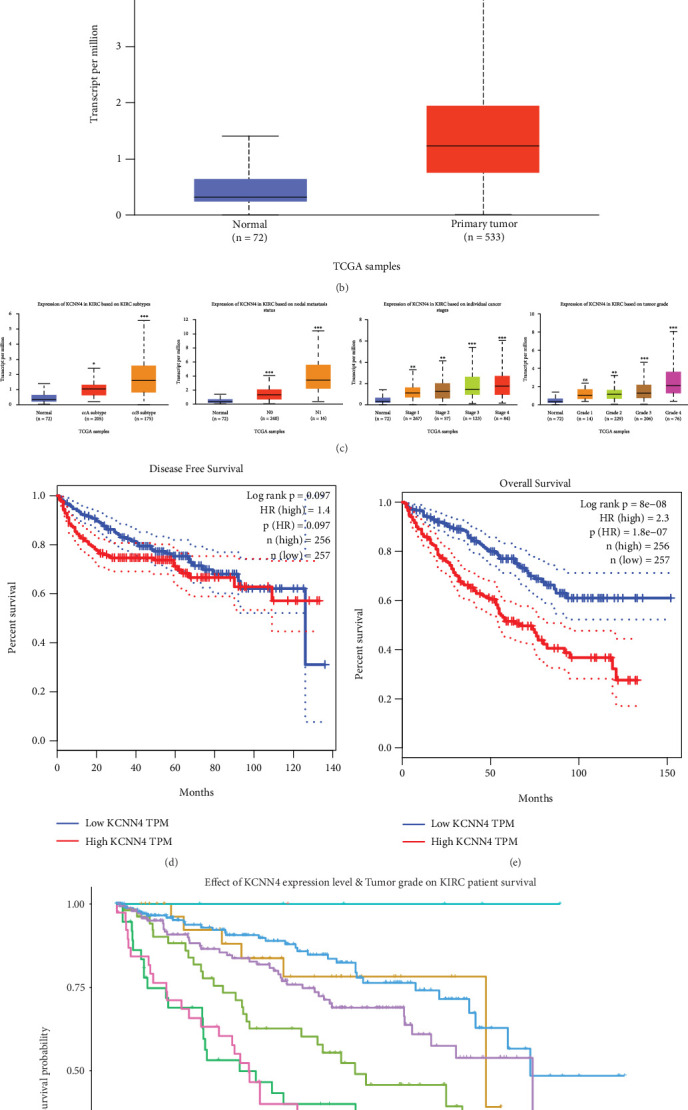
Aberrant expression and strong clinical association of KCNN4 in ccRCC. (a) Pan-cancer analysis of KCNN4 expression in clinical samples from TCGA database. Blue bar: normal tissues; red bar: tumors. (b) Detection of KCNN4 in paired or unpaired ccRCC samples from KIRC database. (c) Detection of KCNN4 expression in ccRCC depending on different tumor subtypes, nodal metastasis, cancer stages, and tumor grades. Disease free survival (DFS) (d) and overall survival (OS) (e) analyses of ccRCC patients from KIRC according to KCNN4 expression. (f) OS analysis of ccRCC patients from KIRC according to KCNN4 expression with tumor grade. Abbreviations: TCGA, The Cancer Genome Atlas; KIRC, kidney renal clear cell carcinoma. ∗*p* < 0.05; ∗∗*p* < 0.01; ∗∗∗*p* < 0.001.

To explore the role of KCNN4 in tumors, TCGA database was first investigated to uncover its expressing level and clinical relevance. Analysis on pan-cancer samples from TCGA showed that KCNN4 overexpressed in tumors compared with normal tissues in most common cancers, including PRAD, LUAD, and SARC (Figure 1(a)). By comparing unpaired ccRCC samples from TCGA, KCNN4 also exhibited distinct overexpression in tumors (Figure 1(b)). Next, tumor malignancy association of KCNN4 was analyzed, and it showed significant differences. First, higher KCNN4 expression was detected in ccB subtype and N1 metastasis compared with ccA subtype and N0 metastasis, respectively. Meanwhile, KCNN4 also displayed an increasing trend of expression along with higher individual cancer stage and tumor grade (Figure 1(c)). At last, KM curves were plotted to demonstrate that higher KCNN4 expression was significantly associated with poorer OS but not DFS of ccRCC patients (Figures 1(d), 1(e), and 1(f)). These results revealed the aberrant expression patterns of KCNN4 and its predominant clinical correlation, which suggested it an indispensable character in tumorigenesis of ccRCC.

### 3.2. Enrichment of KCNN4-Related Genes in Immune Signaling Pathways

**Figure 2:**
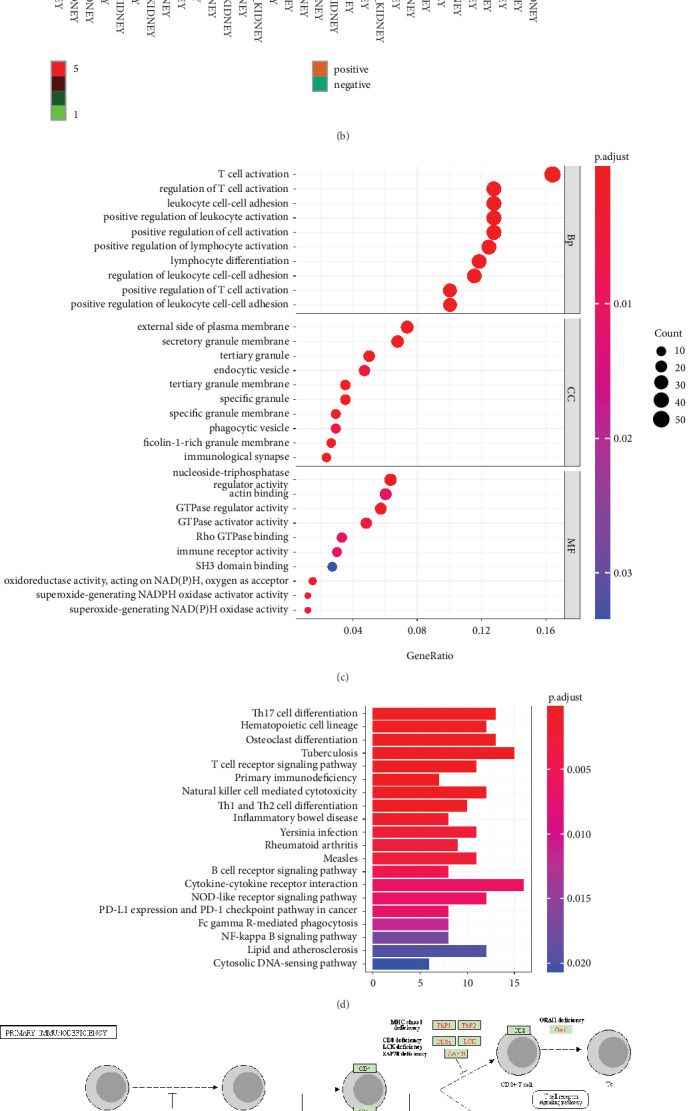
Functional enrichment of KCNN4-related genes in immune-related pathways. Heatmaps of several genes mostly correlated with KCNN4 in cancer tissues (a) and renal carcinoma cell lines (b) from TCGA. (c) Results of GO analysis on all genes correlated with KCNN4 in cancer tissues from TCGA. BP: biological processes; CC: cellular components; MF: molecular function. (d) Results of KEGG analysis on KCNN4-related genes. (e) Overview of the representative immune signaling pathway in which DEGs were enriched. (f) Results of representative KEGG output from GSEA analysis on KCNN4-related genes. Abbreviations: GO, Gene Ontology; KEGG, Kyoto Encyclopedia of Genes and Genomes; GSEA, Gene Set Enrichment Analysis.

**Figure 3:**
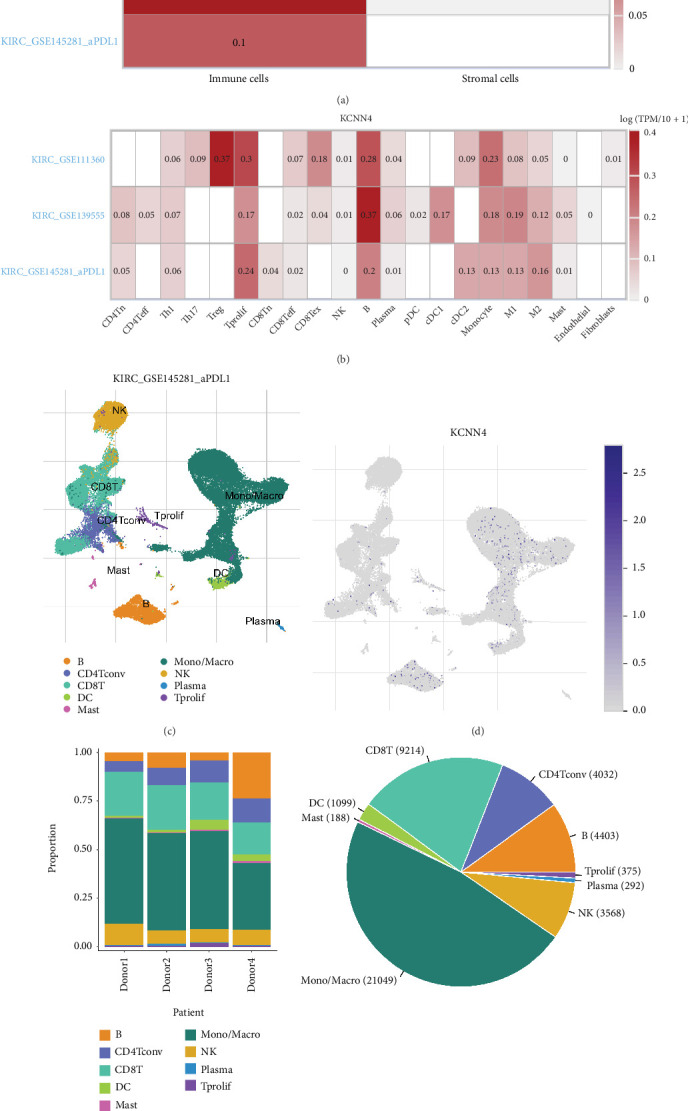
Dominant distribution of KCNN4 in immune cells in TME of ccRCC. (a) Detection of KCNN4 expression in immune cells and stromal cells in three data sets using TISCH database. (b) Detection of KCNN4 expression in single immune cell in three data sets using TISCH database. (c) Single immune cell distribution in ccRCC. (d) KCNN4 distribution in TIM in data set GSE145281_aPDL1. (e) Single cell composition in each (left part) and all (right part) of the four ccRCC patient donors. Abbreviations: TME: tumor microenvironment; TIM: tumor immune microenvironment; TISCH: Tumor Immune Single-Cell Hub.

In total, 363 KCNN4-related genes were collected from tumors of ccRCC samples, and the top 26 of them were shown in the heatmap (Figure 2(a)). Subsequently, these genes were subjected to correlation analysis with KCNN4 in ccRCC cell lines. The top 100 related genes were reserved and top 40 of them were shown in the heatmap as well (Figure 2(b)). After conducting GO and KEGG pathway analyses on these genes, they manifested apparent immune-related features. GO analysis revealed that KCNN4-related genes mainly participated in the BP of immune cells adhesion, including T cell, leukocyte, and lymphocyte (Figure 2(c)). KEGG pathway annotation reached similar results, noting that these genes were closely related to immune responses in vivo (Figure 2(d)). To further illustrate the intimate relationship, an overall review of the primary immunodeficiency (PID) pathway was obtained, and it could be vividly recognized that most of these genes took part in this very process (Figure 2(e)). At last, GSEA results of KEGG were offered as a strong proof (Figure 3(f)). These results helped confirming the abundant enrichment of KCNN4-related genes in immune-related processes and signalings.

### 3.3. Distribution of KCNN4 in Tumor Immune Microenvironment and Proportion of Diverse Cell Types in ccRCC

Inspired by above results, the immune correlation of KCNN4 itself in ccRCC was investigated immediately. First, three independent data sets (GSE111360, GSE139555, and GSE145281_aPDL1) from GEO database were chosen to determine the KCNN4 level in TME. As a result, KCNN4 presented much higher expression in immune cells, compared with the stromal cells (Figure 3(a)). Detailed analysis showed that in three data sets, KCNN4 was mostly enriched in Treg cells, B cells, and proliferative T cells, respectively (Figure 3(b)). Then, data set GSE145281_aPDL1 was further studied in the form of single cell analysis. On one hand, monocytes/macrophages obtained the dominant proportion. On the other hand, the distribution of KCNN4 in diverse types of immune cells manifest its abundance in proliferative T cells, B cells, and monocytes/macrophages (Figures 3(c) and 3(d)). To validate the findings, information of single cell composition from four ccRCC patients was analyzed. Not surprisingly, monocytes/macrophages accounted for the largest proportion in every independent sample and reached nearly half the immune cells in total (*n* = 21,049; Figure 3(e)).

### 3.4. Correlation of KCNN4 with Immune Cell Infiltration and Immunotherapy Markers

**Figure 4:**
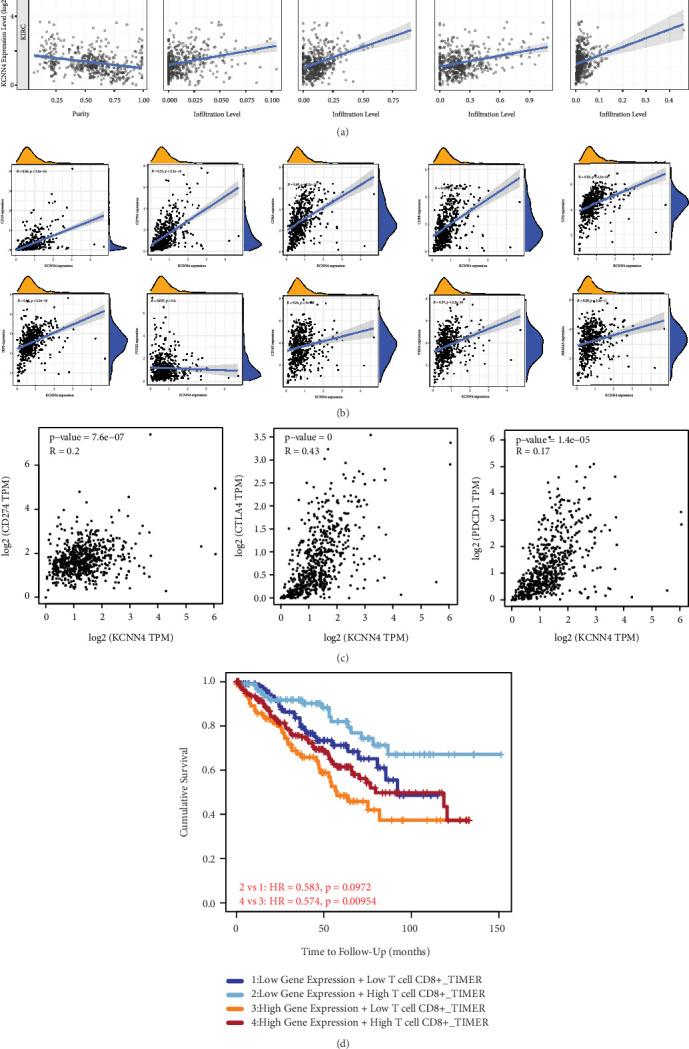
Significant association of KCNN4 with immune cell infiltration. (a) Immune cell infiltration analysis of KCNN4 after adjusted for tumor purity using TIMER database. (b) Spearman's correlation analysis of KCNN4 and various immune cell markers. (c) Correlation analysis of KCNN4 and representative immunotherapy markers. (d) Survival analysis of KIRC patients depending on KCNN4 and CD8+ T cell expression. Abbreviations: TIMER, The Tumor IMmune Estimation Resource.

Given the critical role of KCNN4 in tumor immune microenvironment (TIM), TIMER database was employed to determine the effect of KCNN4 on immune cell infiltration level in ccRCC. After adjusting for purity, infiltration of representative immune cells including Tregs, CD4+ T cells, and CD8+ T cells, along with cancer associated fibroblast, was measured. The results of Spearman's correlation analysis showed the degree of immune cell infiltration increased following the higher expression of KCNN4 (*p* < 0.05; Figure 4(a)). Next, the level of well-established markers (e.g., CD19, CD163, CD79A, and IRF5) of diverse immune cells was detected using the same method. It could be concluded from the fitting curves that KCNN4 expression was significantly positively correlated with immune cell markers level (*p* < 0.05; Figure 4(b)). Meantime, the same analysis was conducted to find relationship between crucial markers of immunotherapy (i.e., immune checkpoint inhibitor therapy) and KCNN4 expression. Surprisingly, it was found that the degree of CD274, PDCD1, and CTLA4 was positively correlated with KCNN4 (*p* < 0.05; Figure 4(c)). At last, KCNN4 gene expression together with CD8+ T cell level was seen as the risk factor for survival analysis on ccRCC patients from TIMER database. The KM curves showed that low KCNN4 expression group did not make significant differences on OS (*p* = 0.097), whereas in high gene expression group, high CD8+ T cells patients gained better survival compared with low CD8+ T cells ones (*p* = 0.0095; Figure 4(d)).

### 3.5. Filtration of Hub miRNA Targeting KCNN4

**Figure 5:**
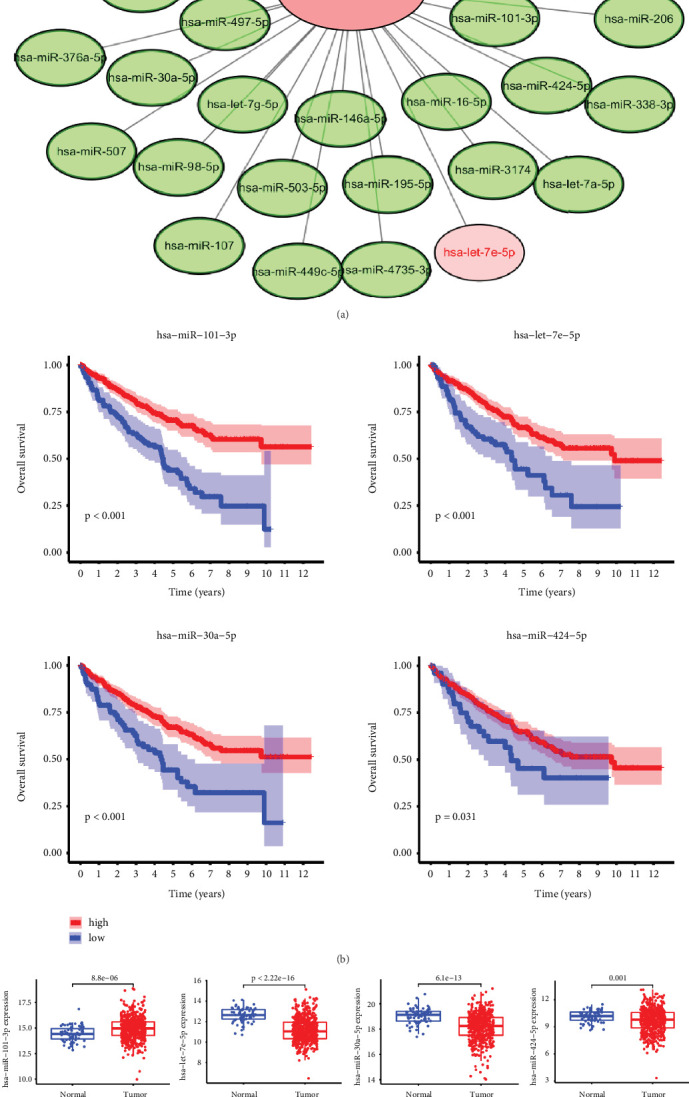
miRNA-let-7e-5p was selected as the hub microRNA. (a) Prediction of the targeted miRNAs using ENCORI database. (b) Four representative miRNAs that were significantly related to OS of ccRCC patients. (c) Detection of four miRNAs expression in KIRC patients. (d) Spearman's correlation analysis of four miRNAs and KCNN4 expression. Abbreviations: ENCORI, The Encyclopedia of RNA Interactomes.

Considering the strong association of KCNN4 with immune signalings which enormously influences cancer progression and treatment strategy, ENCORI database was used to predict miRNAs that directly targets KCNN4 and was meant to discover potential ce-RNA network. As a result, a total of 41 potential miRNAs were picked out (Figure 5(a)), and detailed information of them was listed in Supplemental Table S1. Among them, four miRNAs (miR-101-3p, let-7e-5p, miR-30a-5p, and miR-424-5p) especially displayed significant association with OS of ccRCC patients (*p* < 0.05; Figure 5(b)). Furthermore, ccRCC samples from TCGA were analyzed to determine the miRNAs levels (Figure 5(c)), and Spearman's correlation analysis was conducted to find the expressing correction between them and KCNN4 (Figure 5(d)). The results indicated that four miRNAs were all negatively correlated with KCNN4 level, among which let-7e-5p displayed the most relevant relationship and most significant expression difference. For the reason, miRNA let-7e-5p was chosen for the following research.

### 3.6. Screening for lncRNAs Potentially Sponging Hub miRNA

**Figure 6:**
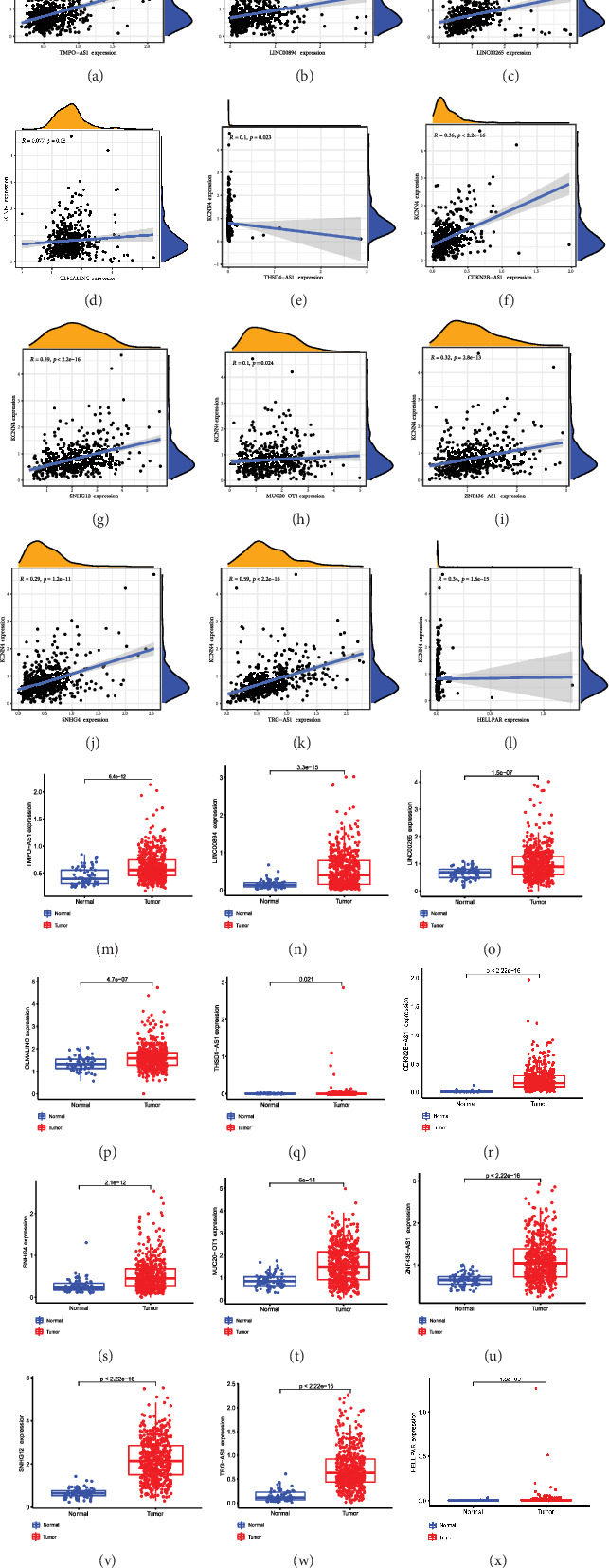
Screening for potential lncRNAs that sponges miRNA-let-7e-5p. (a)–(l) Spearman's correlation analysis of 12 predicted lncRNAs and KCNN4 expression. (m)–(x) Detection of 12 predicted lncRNAs expression in KIRC patients.

After selecting let-7e-5p as hub miRNA which potentially functioned by silencing KCNN4, ENCORI was applied again to discover underlying lncRNAs involved in the functional network. As a result, 12 lncRNA were filtered out and shown in Table 1. Using the same methods as described above, correlation analysis and expression detection were performed on these lncRNAs. As expected, they turned out to be positively correlated with KCNN4 expression and overexpressed in tumors compared with adjacent tissues (Figures 6(a), 6(b), 6(c), 6(d), 6(e), 6(f), 6(g), 6(h), 6(i), 6(j), 6(k), 6(l), 6(m), 6(n), 6(o), 6(p), 6(q), 6(r), 6(s), 6(t), 6(u), 6(v), 6(w), and 6(x)).

### 3.7. Construction of the ce-RNA Network-Based Risk Signature in ccRCC

**Figure 7:**
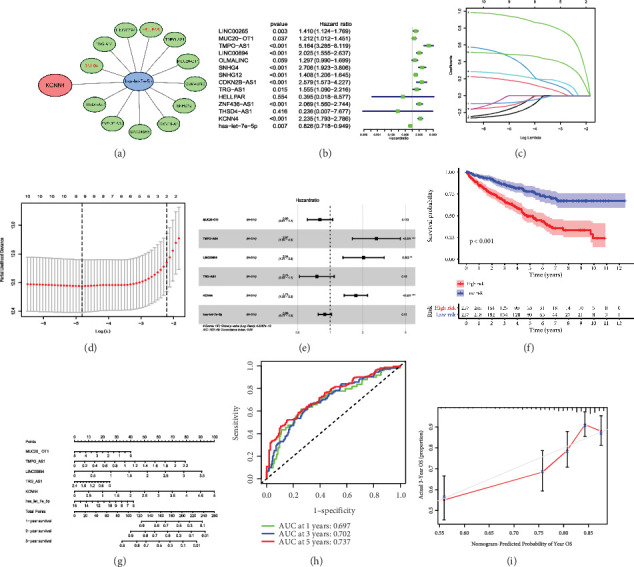
Establishment of the ce-RNA based-gene signature and prognosis model. (a) The heatmap of KCNN4-centred ce-RNA network. (b) Univariate Cox regression analysis of all genes. *p*-Values and HRs were shown. (c) and (d) LASSO regression analysis of all lncRNAs to pick out applicable ones for gene signature. (e) Univariate Cox regression analysis of selected four lncRNAs, together with KCNN4 and miRNA in 514 ccRCC patients from TCGA database. (f) Survival analysis of ccRCC patients from high- and low-risk groups according to the risk scores from gene signature. (g) Nomogram of the prognosis model derived from the risk signature. Survival rate at 1-, 3-, and 5-year from the model was shown as well. (h) ROC curves to validate the sensitivity and specificity of the model. AUC at 1, 3, and 5 years was noted. (i) Calibration curve to validate the predictive efficiency of the model. *Y*-axis represents the actual 3-year OS and *X*-axis represents the predicted 3-year OS. Abbreviations: ROC, receiver operating characteristic; AUC, area under curve.

To further illuminate the significance of the ce-RNA network centering on KCNN4 (Figure 7(a)), a risk-signature was constructed accordingly. First, univariate Cox regression analysis was performed on 12 predicted lncRNAs, along with miRNA-let-7e-5p and KCNN4. The individual *p*-value and HR were shown and depicted in the form of forest plot (Figure 7(b)). In addition to lncRNA HELLPAR and THSD4-AS1, all lncRNAs, with the HR number larger than 1, exhibited significant association with patients' OS (Table 2). Next, all genes were subjected to the LASSO regression analysis and the output provided four powerful prognostic lncRNA, including MUC20-OT1, TMPO-AS1, LINC00894, and TRG-AS1. These genes were then applied together with KCNN4 and miRNA to construct the risk signature, and the risk score for each patient was calculated using the coefficients obtained from the LASSO algorithm (Figures 7(c), 7(d), and 7(e)), which developed the following formula: risk score = −0.219291 × MUC20-OT1 + 0.982848 × TMPO-AS1 + 0.711030 × LINC00894 + (−0.286150) × TRG-AS1 + 0.545779 × KCNN4 + (−0.115140) × hsa-let-7e-5p. According to the median risk score, ccRCC patients in training cohort from TCGA were divided into high-risk (*n* = 257) and low-risk (*n* = 257) groups, and the high-risk patients displayed apparently worse OS compared with the low-risk ones in the survival analysis (*p* < 0.001; Figure 7(f)). Furthermore, the nicely established prognostic model was depicted into the nomogram (Figure 7(g)), and the predictive value was assessed using receiver operating characteristic (ROC) curves and calibration curve. ROC curves showed the respective area under curve (AUC) at 1-, 3-, and 5-year was 0.697, 0.702, and 0.737 (Figure 7(h)). By testing on the validation cohort of kidney renal clear cell carcinoma (KIRC) patients, the calibration curve displayed certain concordance between observed survival probability and the predicted results for 3-year OS (Figure 7(i)). Taken together, these results suggested that the ce-RNA network-based risk signature held considerable prognostic efficiency and clinical value for ccRCC patients.

### 3.8. Further Evaluation of Prognostic Models and the Significance of KCNN4 in Clinical Pharmacology

**Figure 8:**
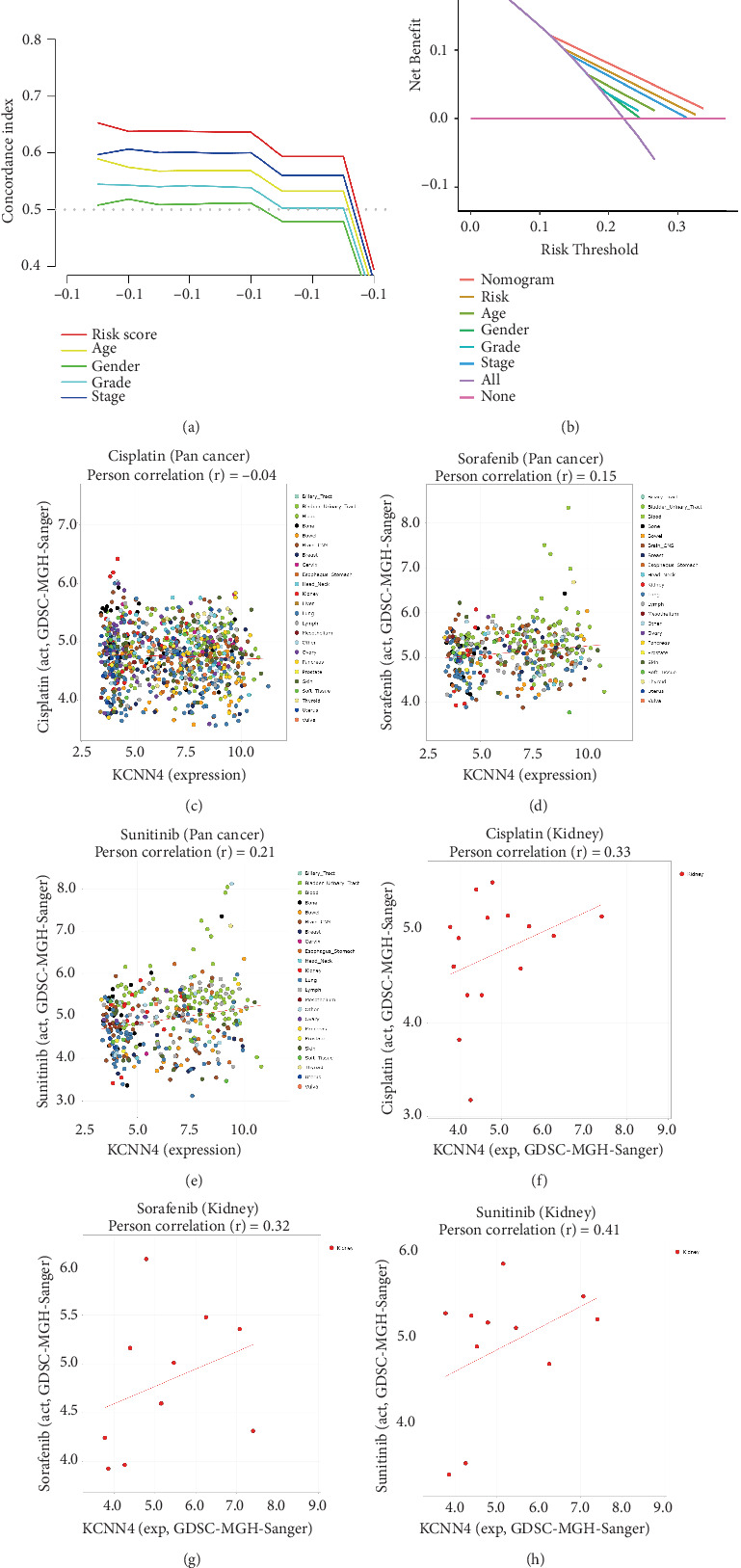
Evaluation of prognostic models and the clinical pharmacological significance of KCNN4. (a) Concordance index curve is used to evaluate the prognostic model. (b) DCA are used to evaluate prognostic models. (c)–(e) The relationship between the expression level of KCNN4 in pan-cancer and the drug sensitivity of cisplatin, sorafenib, and sunitinib. (f)–(h) The relationship between the expression level of KCNN4 in renal carcinoma and the drug sensitivity of cisplatin, sorafenib, and sunitinib.

Concordance index curve (Figure 8(a)) and DCA (Figure 8(b)) were established to evaluate prognostic models based on KCNN4-associated ceRNA networks. The higher the AUC, the higher the accuracy of the corresponding index in predicting clinical prognosis. As shown in Figure 8, the AUC corresponding to risk score or nomogram is the highest. This indicated that the prognostic model we constructed has high efficiency. In addition, we analyzed the relationship between the expression level of KCNN4 and the sensitivity of cisplatin, sorafenib, and sunitinib. The results showed that the expression level of KCNN4 in pan-cancer had a Pearson correlation of −0.04, 0.15, and 0.21 with cisplatin (Figure 8(c)), sorafenib (Figure 8(d)), and sunitinib (Figure 8(e)), respectively. The corresponding Pearson correlations in kidney cancer were 0.33 (Figure 8(f)), 0.32 (Figure 8(g)), and 0.41 (Figure 8(h)), respectively. The above results indicated that the total expression of KCNN4 in pan-cancer was not strongly correlated with the sensitivity of these three drugs. However, there is a certain positive correlation in renal cancer, that is, the higher the expression of KCNN4, the higher the drug sensitivity.

### 3.9. Prominent Tumor-Promoting Role of KCNN4 in ccRCC Cells

**Figure 9:**
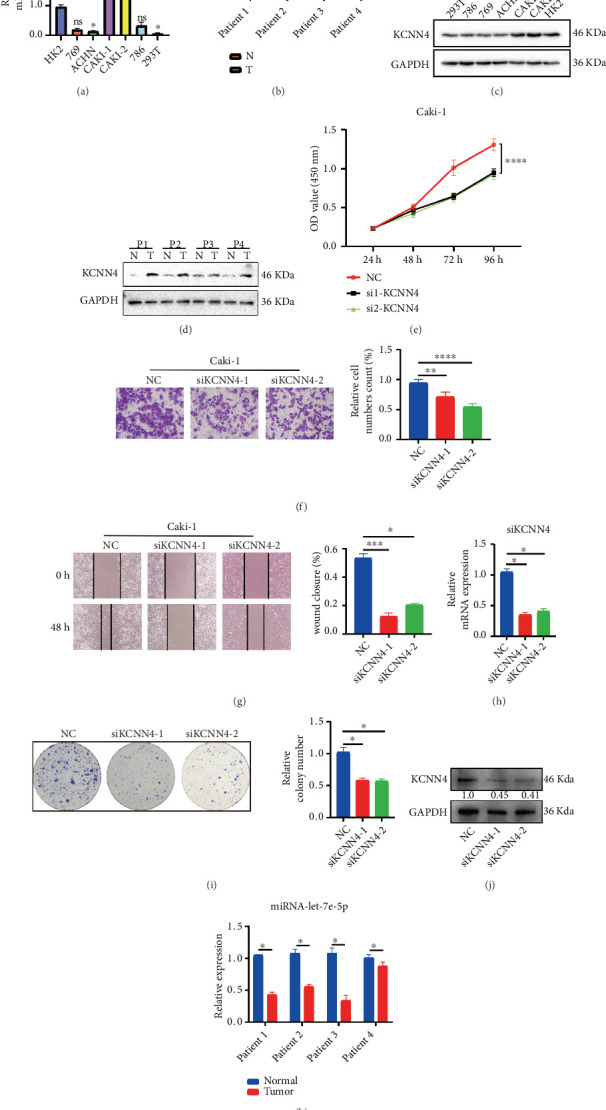
In vitro oncogenic role of KCNN4 in ccRCC. (a) and (b) Results of qRT-PCR to detect KCNN4 mRNA level in ccRCC cell lines and four clinical samples. (c) and (d) Results of western blot to detect KCNN4 protein level in ccRCC cell lines and four clinical samples. (e) Result of CCK8 assay after transfecting siRNAs into Caki-1 cells. (f) Transwell assay after transfecting siRNAs into Caki-1 cells. (g) Wound healing assay after transfecting siRNAs into Caki-1 cells. (h) Knockdown efficiency verified by qRT-PCR. (i) Colony formation assay after transfecting siRNAs into Caki-1 cells. (j) Knockdown efficiency verified by western blot (relative gray value of KCNN4 is presented under its band). (k) Results of qRT-PCR detection of hub micRNA let-7e-5p expression in four paired KIRC samples ∗*p* < 0.05; ∗∗*p* < 0.01; ∗∗∗*p* < 0.001. Abbreviations: siRNA, small interfering RNA.

Following the comprehensive bioinformatics analysis of KCNN4 in ccRCC, solid fundamental experiments were performed to validate the inspiring findings. At the beginning, detection of KCNN4 expression was conducted using qRT-PCR and western blot. It was found that in five ccRCC cell lines (i.e., 769-P, ACHN, Caki-1, Caki-2, and 786-O), KCNN4 mRNA expression was significantly elevated in Caki-1 and Caki-2 cells (Figure 9(a)), with its protein level consistent in Caki-1 and Caki-2 as well (Figure 9(c)). In paired samples from four clinically diagnosed ccRCC patients, both mRNA and protein relative expression were significantly higher in tumors compared with normal tissues (Figures 9(b) and 9(d)). Next, Caki-1 cells were chosen for further research by knocking down KCNN4 through transfecting siRNAs. Result of CCK8 assay showed that KCNN4 silencing profoundly undermined the proliferative ability of Caki-1 cells (Figures 9(e)). Transwell and wound healing assays indicated that knockdown of KCNN4 also remarkably impaired cells' capabilities of invasion, migration, and colony formation assay (Figures 9(f), 9(g), and 9(i)). The knockdown efficiency of KCNN4 was verified by qRT-PCR and western blot (Figures 9(h) and 9(j)). Besides, qRT-PCR detection of hub miRNA let-7e-5p showed relatively lower expression in tumor tissue compared with normal tissue (Figure 9(k)). These basal in vitro experiments solidly certified previous findings and helped establishing the prominent oncogenic role of KCNN4 in ccRCC.

## 4. Discussion

As a critical calcium-activated potassium channel protein, KCNN4 regulated diverse basal molecular and biological events in cells, and it had been increasingly studied in cancer field. For the first time of thorough research on KCNN4 in cancer, Jiang et al. found that gamma-aminobutyric acid type A receptor could interact with KCNN4 to induce Ca^2+^ entry, which led to the activation of nuclear factor *κ*B signaling and ultimately facilitated macrophage infiltration [18]. This process was considered important for macrophage recruitment and tumor progression in pancreatic cancer. Likewise, the significance of KCNN4 was also well investigated in many types of malignant tumor. However, it has not yet been fully studied in RCC, for which reason we conducted this research.

In the present study, KCNN4 was confirmed as a crucial gene that enormously affected clinical prognosis of ccRCC patients. Using multiple methods, we mainly explored the intimate relationship between KCNN4 and the status of TIM, and successfully established the prognosis model derived from KCNN4-centred ce-RNA network. Combined with solid fundamental experiments, it was the first time for KCNN4 to be deeply studied in ccRCC. To begin with, we used KIRC database from TCGA to detect KCNN4 level in different types and stages of cancers, and the aberrant expressing pattern turned out to be similar in most cancer types. It is worth mentioning that KCNN4 expression exhibited the same trend with tumor stages and grades in ccRCC, indicating the potential direct relationship between KCNN4 and tumor malignancy. The result of survival analysis also showed that KCNN4 was a vital risk factor to impair patients' long-term prognosis. These findings preliminarily built the tight association between KCNN4 and tumor progression of ccRCC.

According to previous studies, KCNN4 could influence tumor derivation, progression, and occurrence in many ways. Using the method of RNA-sequence, Mo et al. reported that KCNN4-mediated Ca^2+^/MET/AKT axis was essential and promoted pancreatic ductal adenocarcinoma cells to proliferate and migrate [11]. Similarly, Li et al. reported that KCNN4 promotes tumor invasion and metastasis through the MAPK/ERK pathway in hepatocellular carcinoma [15]. Interestingly, Xu et al. found that KCNN4 could boost the progression of lung adenocarcinoma by synchronously activating the AKT and ERK signaling pathways [19], which granted their studies certain similarity and repeatability. Our study, however, focused on immune-related analyses and proved that KCNN4 could make intimate connections to immune signalings and immunotherapy, which could probably help understanding the mechanisms of KCNN4 affecting tumorigenesis. By analyzing DEGs based on KCNN4 level, their enrichment was observed in immune-related signaling pathways, represented by PID. According to Thaventhiran et al., PID is characterized by recurrent autoimmunity and cancer, and chances are lymphoid malignancies [20]. Results of single-cell analysis showed the abundant distribution of KCNN4 in B cells, monocytes/macrophages, and proliferative and regulative T cells. While immune cell infiltration analysis revealed the strong positive correlation between immune responses and KCNN4 itself. Besides, the level of immunotherapy markers was also considered significantly responding to KCNN4. Similarly, Chen et al. also found that KCNN4 had significant correlation with tumor-infiltrating immune cells and affected the TME immune status in ccRCC [21]. These findings proved the sensitive and effective immune responses of organism to KCNN4 activities, which could be the target of immune therapies for ccRCC patients in the future.

miRNAs are a kind of ncRNAs well known to play imperative roles in tumor BP, functioning by silencing the targeted mRNA [22]. lncRNAs are a set of non-coding transcripts longer than 200 nucleotides, which have been shown to be indispensable in the multifarious regulation of mRNAs [23]. The ce-RNA network is a collective pool of transcripts that synergistically insulate miRNA activity through competition for the same individual miRNA, thus formulating the internal regulatory relationship of RNA [24]. It has been proved that lncRNAs could serve as the critical ce-RNA to sponge correlated with miRNAs, formulating an lncRNA–miRNA–mRNA regulatory network, to fulfill the derepression of downstream genes [25–27]. In this situation, the interaction between lncRNA and mRNA through contacting with same miRNA is called endogenous competition, which has been proved to widely operate in the origination and progression of tumor in RCC [28, 29]. In our study, we successively screened for potential miRNAs and competitive lncRNAs with the goal of KCNN4. After correlation analysis and expression detection, the well-performed hub miRNA-let-7e-5p and four lncRNAs were chosen for the construction of gene signature. To our relief, the final prognosis model based on the signature exhibited respectable prognostic efficiency and reliability. Notably, our work solidly supported the prognostic value and potential applicable value of ce-RNA network in clinical work.

Previous study from Chen et al. mainly introduced the crosstalk between KCNN4 and TIM, confirming that KCNN4 enormously affected the immune status of tumor and the prognosis of ccRCC patients [21]. Meantime, our work accomplished parallel work and made additional research on related ce-RNA to construct a more reliable gene signature. We also performed functional experiments on KCNN4 to demonstrate its oncogenic role of in ccRCC cells. Overall, we made a comprehensive study on KCNN4 to prove its critical role in promoting the tumor progression and affecting the long-term prognosis of ccRCC patients. Besides, there certainly were shortages in our study. First, basal analyses were all conducted on patients from TCGA database, whereas the experimental validation seem to be in short of clinical samples. Besides, there was no measurement of immune-related markers in vitro or in vivo to validate the significant immune correlation of KCNN4. Moreover, no assays were performed to verify the related lncRNAs and miRNA, which made the ce-RNA network less convincing. Ultimately, supplementary research on KCNN4 is essentially needed, and more dependable conclusion should be achieved in the future.

## 5. Conclusion

Our work found the aberrant expression and prominent clinical correlation of KCNN4 in ccRCC through online databases. Multiple methods of bioinformatics analyses and fundamental experiments confirmed the obvious role of KCNN4 in promoting tumor progression in vitro. The ce-RNA network based-risk signature exhibited satisfactory efficiency. Therefore, we conclude that KCNN4 may serve as a potential biomarker for the prognosis and immunotherapy effect of ccRCC patients in the future.

## Figures and Tables

**Table 1:** Underlying lncRNAs involved in the functional network.

lncRNA	miRNA	Cor	*p*-Value	logFC	Diff *p*-Value
HELLPAR	hsa-let-7e-5p	−0.2531	5.35 × 10^−9^	0.0082	1.81 × 10^−9^
SNHG4	hsa-let-7e-5p	−0.2479	1.11 × 10^−8^	0.2573	2.06 × 10^−12^
TRG-AS1	hsa-let-7e-5p	−0.1575	0.0003	0.5680	1.79 × 10^−35^
TMPO-AS1	hsa-let-7e-5p	−0.1548	0.0004	0.1917	6.36 × 10^−12^
LINC00894	hsa-let-7e-5p	−0.1427	0.0011	0.3782	3.31 × 10^−15^
LINC00265	hsa-let-7e-5p	−0.1340	0.0023	0.3415	1.50 × 10^−7^
OLMALINC	hsa-let-7e-5p	−0.1261	0.0041	0.2780	4.66 × 10^−7^
THSD4-AS1	hsa-let-7e-5p	−0.1163	0.0081	0.0100	0.0211
CDKN2B-AS1	hsa-let-7e-5p	−0.1150	0.0089	0.2028	2.56 × 10^−40^
SNHG12	hsa-let-7e-5p	−0.1137	0.0096	1.5403	2.73 × 10^−37^
MUC20-OT1	hsa-let-7e-5p	−0.1091	0.0131	0.7238	5.98 × 10^−14^
ZNF436-AS1	hsa-let-7e-5p	−0.1001	0.0228	0.4571	5.44 × 10^−18^

**Table 2:** lncRNA which are significantly associated with patients' OS.

Id	HR	HR.95L	HR.95H	*p*-Value
LINC00265	1.4099	1.1238	1.7688	0.0030
MUC20-OT1	1.2116	1.0118	1.4508	0.0368
TMPO-AS1	5.1643	3.2847	8.1194	1.15 × 10^−12^
LINC00894	2.0249	1.5548	2.6372	1.66 × 10^−7^
OLMALINC	1.2971	0.9902	1.6990	0.0590
SNHG4	2.7058	1.9234	3.8064	1.09 × 10^−8^
SNHG12	1.4081	1.2055	1.6447	1.57 × 10^−5^
CDKN2B-AS1	2.5791	1.5735	4.2273	0.0002
TRG-AS1	1.5546	1.0905	2.2163	0.0147
HELLPAR	0.3950	0.0182	8.5766	0.5542
ZNF436-AS1	2.0688	1.5600	2.7436	4.48 × 10^−7^
THSD4-AS1	0.2359	0.0072	7.6766	0.4163
KCNN4	2.2353	1.7932	2.7864	8.43 × 10^−13^
hsa-let-7e-5p	0.8259	0.7183	0.9495	0.0072

## Data Availability

The original contributions presented in the study are included in the article/Supplementary Material. Further inquiries can be directed to the corresponding author.

## Abbreviations

RCC: Renal cell carcinoma
ccRCC: Clear cell renal cell carcinoma
DEG: Differentially expressed genes
GEO: Gene Expression Omnibus
GSEA: Gene Set Enrichment Analysis
OS: Overall survival
TCGA: The Cancer Genome Atlas
FBS: Fetal bovine serum
KEGG: Kyoto Encyclopedia of Genes and Genomes.

## Supplementary Materials

Supplementary Materials Table S1. Predicted miRNAs that directly targets KCNN4
