# Supplementary material for: Development of a Novel KCNN4-Related ceRNA Network and Prognostic Model for Renal Clear Cell Carcinoma
Source: Anal Cell Pathol (Amst). 2023 Jan 24;2023:2533992. doi: 10.1155/2023/2533992 (PMC11401688; doi:10.1155/2023/2533992)
Supplement: Supplementary Materials — Table S1. Predicted miRNAs that directly targets KCNN4 [file 2533992.f1.docx]

Table S1

| Gene | miRNA | cor | pvalue | logFC | diffPval |
| --- | --- | --- | --- | --- | --- |
| KCNN4 | hsa-miR-101-3p | -0.1663 | 0.0001 | 0.5165 | 8.81E-06 |
| KCNN4 | hsa-let-7e-5p | -0.1627 | 0.0002 | -1.4567 | 7.36E-22 |
| KCNN4 | hsa-miR-30a-5p | -0.1400 | 0.0014 | -0.8699 | 6.08E-13 |
| KCNN4 | hsa-miR-424-5p | -0.1076 | 0.0144 | -0.4865 | 0.0010 |
| KCNN4 | hsa-miR-503-5p | -0.0999 | 0.0232 | -0.5160 | 0.0013 |
| KCNN4 | hsa-let-7a-5p | -0.0872 | 0.0475 | -0.5963 | 5.16E-07 |
| KCNN4 | hsa-miR-98-5p | -0.0820 | 0.0624 | -0.3626 | 8.20E-05 |
| KCNN4 | hsa-let-7f-5p | -0.0805 | 0.0675 | -1.2635 | 1.66E-10 |
| KCNN4 | hsa-miR-338-3p | -0.0797 | 0.0701 | 0.3816 | 0.0033 |
| KCNN4 | hsa-let-7b-5p | -0.0694 | 0.1149 | 0.6179 | 5.03E-09 |
| KCNN4 | hsa-miR-107 | -0.0291 | 0.5090 | -0.2095 | 0.0062 |
| KCNN4 | hsa-miR-1343-3p | -0.0248 | 0.5734 | -0.4635 | 1.00E-06 |
| KCNN4 | hsa-miR-129-2-3p | -0.0233 | 0.5975 | -1.0893 | 9.21E-19 |
| KCNN4 | hsa-let-7g-5p | -0.0042 | 0.9239 | -1.2134 | 2.17E-24 |
| KCNN4 | hsa-miR-29c-3p | 0.0078 | 0.8588 | -0.5265 | 8.48E-07 |
| KCNN4 | hsa-miR-4735-3p | 0.0136 | 0.7582 | 0.0018 | 0.7220 |
| KCNN4 | hsa-miR-224-5p | 0.0178 | 0.6857 | 2.2798 | 5.14E-28 |
| KCNN4 | hsa-miR-497-5p | 0.0219 | 0.6190 | -0.0142 | 0.8354 |
| KCNN4 | hsa-miR-195-5p | 0.0231 | 0.6004 | 0.0428 | 0.6641 |
| KCNN4 | hsa-miR-449c-5p | 0.0271 | 0.5381 | -0.0137 | 0.4916 |
| KCNN4 | hsa-miR-24-3p | 0.0332 | 0.4516 | -0.1033 | 0.1442 |
| KCNN4 | hsa-miR-654-5p | 0.0385 | 0.3823 | -0.3034 | 0.0102 |
| KCNN4 | hsa-miR-15a-5p | 0.0404 | 0.3587 | 0.9995 | 2.15E-19 |
| KCNN4 | hsa-miR-206 | 0.0511 | 0.2465 | -3.3693 | 3.53E-46 |
| KCNN4 | hsa-miR-16-5p | 0.0579 | 0.1889 | 0.8993 | 2.81E-18 |
| KCNN4 | hsa-miR-3174 | 0.0601 | 0.1728 | 0.1501 | 0.0080 |
| KCNN4 | hsa-miR-103a-3p | 0.0643 | 0.1445 | 0.2384 | 0.2079 |
| KCNN4 | hsa-miR-147a | 0.0646 | 0.1424 | 0.0018 | 0.7220 |
| KCNN4 | hsa-let-7i-5p | 0.0685 | 0.1200 | 0.5064 | 1.84E-06 |
| KCNN4 | hsa-miR-520c-3p | 0.0700 | 0.1121 | 0.0235 | 0.2765 |
| KCNN4 | hsa-miR-374a-5p | 0.0805 | 0.0673 | 0.3366 | 0.0005 |
| KCNN4 | hsa-miR-1-3p | 0.0888 | 0.0436 | -1.9504 | 5.48E-21 |
| KCNN4 | hsa-miR-507 | 0.0888 | 0.0435 | -1.3156 | 8.72E-42 |
| KCNN4 | hsa-miR-22-3p | 0.0955 | 0.0299 | -0.1209 | 0.0457 |
| KCNN4 | hsa-miR-200c-3p | 0.1020 | 0.0204 | -5.6472 | 1.26E-36 |
| KCNN4 | hsa-miR-15b-5p | 0.1135 | 0.0098 | 0.2830 | 0.0187 |
| KCNN4 | hsa-miR-296-5p | 0.1408 | 0.0013 | -0.1005 | 0.7812 |
| KCNN4 | hsa-miR-3619-5p | 0.1441 | 0.0010 | 0.0807 | 0.0840 |
| KCNN4 | hsa-miR-7-5p | 0.1487 | 0.0007 | 0.1973 | 0.1653 |
| KCNN4 | hsa-miR-376a-5p | 0.1508 | 0.0006 | -1.2151 | 2.01E-19 |
| KCNN4 | hsa-miR-146a-5p | 0.2902 | 0.0000 | 0.9619 | 1.95E-10 |

predicted miRNAs that directly targets KCNN4
